# Identifying Dynamic Protein Complexes Based on Gene Expression Profiles and PPI Networks

**DOI:** 10.1155/2014/375262

**Published:** 2014-05-18

**Authors:** Min Li, Weijie Chen, Jianxin Wang, Fang-Xiang Wu, Yi Pan

**Affiliations:** ^1^School of Information Science and Engineering, Central South University, Changsha 410083, China; ^2^Department of Mechanical Engineering, University of Saskatchewan, SK, Canada S7N 5A9; ^3^Department of Computer Science, Georgia State University, Atlanta, GA 30302-4110, USA

## Abstract

Identification of protein complexes from protein-protein interaction networks has become a key problem for understanding cellular life in postgenomic era. Many computational methods have been proposed for identifying protein complexes. Up to now, the existing computational methods are mostly applied on static PPI networks. However, proteins and their interactions are dynamic in reality. Identifying dynamic protein complexes is more meaningful and challenging. In this paper, a novel algorithm, named DPC, is proposed to identify dynamic protein complexes by integrating PPI data and gene expression profiles. According to Core-Attachment assumption, these proteins which are always active in the molecular cycle are regarded as core proteins. The protein-complex cores are identified from these always active proteins by detecting dense subgraphs. Final protein complexes are extended from the protein-complex cores by adding attachments based on a topological character of “closeness” and dynamic meaning. The protein complexes produced by our algorithm DPC contain two parts: static core expressed in all the molecular cycle and dynamic attachments short-lived. The proposed algorithm DPC was applied on the data of *Saccharomyces cerevisiae* and the experimental results show that DPC outperforms CMC, MCL, SPICi, HC-PIN, COACH, and Core-Attachment based on the validation of matching with known complexes and hF-measures.

## 1. Introduction

In the postgenomic era, more and more attention has been paid to proteomics. Proteins are central part of life activity. Within a cell, proteins cannot work alone to carry out cellular functions while these cellular functions are performed by many proteins bound together into protein complexes [1]. With the development of high-throughput techniques, amount of protein-protein interactions (PPI) has been catalogued. Such protein-protein interaction data can provide us with a chance to understand complicated biological systems from a network view.

Up to now, many computational methods have been proposed for identifying protein complexes from PPI networks. The most common network-based methods are to detect dense subgraphs from PPI networks as complexes for the researchers believe that proteins in the same complex generally implement the same or similar function and tend to interact with each other [2, 3]. Spirin and Mirny [2] proposed to enumerate all the maximal cliques (fully connected subgraphs) as protein complexes. Liu et al. [4] presented a method called CMC (Clustering-based on Maximal Cliques) which also identifies protein complexes based on maximal cliques. The maximal cliques are weighted and the highly overlapping cliques are merged or removed. Palla et al. [5] proposed a clique percolation method, named CPM, to identify overlapping communities in complex networks. Our group also proposed a clique-based method IPC-MCE [6] which detects maximal cliques first and then extends from the maximal cliques to generate protein complexes. MCODE proposed by Bader and Hogue [7] is a local-searched method to detect protein complexes based on the proteins' connectivity values in PPI network. Altaf-UI-Amin et al. [8] gave an algorithm DPClus based on the combination of density and peripheral proteins to mine densely connected subgraphs. By modifying the DPClus algorithm based on new topological structures, our group proposed a new method named IPCA [9] to identify dense subgraphs as protein complexes. Ding et al. [10] detected dense subgraphs by using minimum vertex cuts on PPI network. Chen et al. [11] introduced a novel method using cliques as seeds and graph entropy. Wang et al. [12] presented a topological algorithm named HKC to predict overlapping clusters by the definition of highest *k*-score and cohesion.

Besides these density-based algorithms, there are some algorithms available to detect protein complexes based on other topological structure. Girvan and Newman [13] developed an algorithms named G-N to detect community structure in complex network by hierarchy division. Luo et al. [14] modified the definition of modules by extending the concept of degree from vertex to subgraphs and developed an agglomerative algorithm MoNet to detect dense subgraphs and other topology subgraphs. We presented an algorithm named HC-PIN [15] to generate protein complexes by using edges clustering coefficient from both weighted and unweighted graphs. More protein complex discovery algorithms can be referred to in [16, 17]. Although different types of clustering algorithms have their own advantages, these algorithms based on dense subgraphs have much better performance than those based on other topological structure.

Recently, some researchers investigated the inherent organization of protein complexes on the basis of topological analysis. Dezso et al. [18] studied the inherent organization of experimentally detected protein complexes of* Scaccharomves cerevisiae* from the data of Gavin et al. 2002 [19] and Ho et al. 2002 [20]. They illustrated that protein complexes of* Saccharomyces cerevisiae* generally contain a core in which proteins are highly coexpressed and share identical functional classification and cellular localization. The protein-complex core is often surrounded by some attachments, a functionally mixed group of proteins, which assist the core to perform subordinate functions. Gavin et al. [21] provided a genome-wide bioinformatics analysis for yeast complexes and demonstrated a similar architecture that protein complexes are comprised of core and additional attachment proteins or protein modules. Wu et al. [22] and Leung et al. [23] explored the Core-Attachment structures of protein complexes from a topological view. Wu et al. [22] detected dense subgraphs as protein-complex cores and Leung et al. [23] proposed a statistical framework to generate protein-complex cores. They both detected the attachments for each core based on the idea of majority rule that proteins connecting with at least half of the proteins in the core will be considered as attachments.

The investigation of inherent organization of protein complexes provides a new clue to identify protein complexes from PPI network. There is a common perspective that proteins in the core tend to interact with each other and are generally highly coexpressed. Dezso and Gavin's work shows that attachments are often short-lived proteins. From this perspective, the protein complex discovery methods based on static PPI networks have limitations. In reality, cellular process is dynamic; proteins and interactions between proteins vary with time.

Considering that gene expression data provide the dynamic expression information for each protein in the cell cycles [24–26], we propose a new Core-Attachment method (DPC) for identifying dynamic protein complex by integrating the gene expression profile with PPI networks. Not only is the topological character considered but also dynamic meaning in DPC. The protein complexes produced by our algorithm DPC contain two parts: static core expressed in all the molecular cycle and dynamic attachments short-lived. The proposed algorithm DPC operates in three phases: detecting protein-complex cores, generating potential dynamic complexes, and filtering false positive complexes. The proposed algorithm DPC was applied on the data of* Saccharomyces cerevisiae*. The experimental results show that DPC can predict more accurate cores or complexes than COACH [22] and Core-Attachments [23] and outperforms CMC [4], MCL [27], SPICi [28], and HC-PIN [15] on the validation of matching with known complexes and hF-measure.

## 2. Algorithm DPC

Different from traditional network-based Core-Attachment methods, the proposed algorithm DPC develops new strategies to identify protein-complex cores and their attachments. The proposed algorithm DPC operates in three phases: detecting protein-complex cores, generating potential dynamic complexes, and filtering false positive complexes. Before the details of the proposed algorithm DPC are introduced, some related definitions are given first.

A protein-protein interaction (PPI) network is modeled as an undirected graph *G* = (*V*, *E*), where *V* represents the set of proteins and an edge (*v*_*i*_, *v*_*j*_) ∈ *E* if and only if protein *v*_*i*_ is found to interact with protein *v*_*j*_. Given such a graph *G*, the degree of a node *v* ∈ *V*, marked as deg⁡(*v*), is defined as the number of neighbors of *v* in *G*. Let *N*_*v*_ denote the set of neighbors of *v*; deg⁡(*v*) = |*N*_*v*_|. 

For a given subgraph *C*⊆*G*, let *V*_*C*_ represent the set of proteins in *C* and *E*_*C*_ denote the set of interactions between two distinct proteins in *C*. The density of *C*, denoted as den(*C*), is defined as the number of edges in it, divided by the number of potential edges; that is,

(1) $$\begin{array}{c} \text{den}\left(C\right)=\frac{2\times \left|E_{C}\right|}{\left|V_{C}\right|\times \left(\left|V_{C}\right|-1\right)}. \end{array}$$

For a protein *v*, its gene expression profile can be abstracted into Ge(*v*) = {*g*(*v*, 1), *g*(*v*, 2),…, *g*(*v*, *k*), …, *g*(*v*, *s*)}, where *g*(*v*, *k*) denotes the expression value of protein *v* in *k*th time course. A protein is said to be active in *k*th time course if its *g*(*v*, *k*) is larger than or equal to a given threshold *T*_*g*_. In this paper, *T*_*g*_ is set as 0.7 which was typically used in previous studies [29]. The proteins in the PPI network which are always active in the cell cycle are called* always active proteins*. *P*_*A*_ is used to describe the set of the always active proteins. For a given protein *v* ∈ *P*_*A*_, *N*_*v*_^*P*_*A*_^ is used to represent the subset of *v*'s neighbor proteins which are always active in the cell cycle; that is, *N*_*v*_^*P*_*A*_^ = *N*_*v*_∩*P*_*A*_. The rest neighbor proteins of the node *v* are collected into a set which is marked as *N*_*v*_^*R*^. It is obvious that *N*_*v*_ is the union of *N*_*v*_^*P*_*A*_^ and *N*_*v*_^*R*^; that is, *N*_*v*_ = *N*_*v*_^*P*_*A*_^ + *N*_*v*_^*R*^.

### 2.1. Detecting Protein-Complex Cores

It has been shown that a protein-complex core is a small group of proteins which are highly coexpressed, share high degree of functional similarity, and have more interactions between themselves [18]. The protein-complex cores, as the key functional units of protein complexes, largely determine the cellular role and essentiality of the corresponding complexes [18]. From a topological view, proteins in the core often have many interacting partners and protein-complex cores often correspond to small and dense subgraphs in a PPI network [16, 17]. From a biological perspective, proteins in the core are generally highly coexpressed and surrounded by a functionally mixed group of proteins, which likely represent short-lived or spurious attachments [18].

According to these properties of protein-complex cores, we first give its definition. A subgraph *C*⊆*G* is called a protein-complex core if it satisfies the following two constraints: (1) all its vertices are always active proteins, that is, for any vertex *v* ∈ *C* it is a vertex of *P*_*A*_ and (2) it is a dense subgraph of *G* (validated with density den(*C*) and the density threshold *T*_*d*_ is set as 0.7 in this paper according to the typical threshold value used in previous works [8, 22]).

Based on the above definition, detecting possible protein-complex cores is divided into two main steps: searching always active proteins and forming possible protein-complex cores. Forming possible protein-complex cores is aimed to group always active proteins into many connected subgraphs according to the topological and dynamic features of protein-complex cores. The description of subroutine for detecting possible protein-complex cores is shown in Algorithm 1.

Firstly, the always active proteins are chosen as candidates of core proteins by evaluating the expression values of each protein in the cell cycle. For an always active protein *v* ∈ *P*_*A*_, its neighbors which also belong to *P*_*A*_ are called its always active neighbors. The core candidate proteins are sorted by the numbers of their own always active neighbors in nonincreasing order. If two vertices *u* and *v* have the same number of always active neighbors (i.e., |*N*_*u*_^*P*_*A*_^ | = |*N*_*v*_^*P*_*A*_^|), they will be secondly sorted by the numbers of their rest neighbors in *G* in descending order. The sorted always active proteins will be stored into a queue *Q*. The first vertex in the queue *Q* is picked and used as a seed to grow a new possible protein-complex core. Once a protein-complex core is completed, all vertices in it will be tagged with “1” and cannot be extended into any other protein-complex cores. The subroutine will stop when the queue *Q* is empty.

Before generating the final protein-complex core, for a seed vertex picked from the queue *Q*, we first look for its corresponding preliminary core. The preliminary core is composed of a seed vertex and its always active neighbors. Note that the neighbors that have been tagged with “1” will not be included. Since proteins in the protein-complex core have relatively more interactions between them, the protein-complex cores should be densely connected subgraphs. Here, a final protein-complex core is generated by removing vertices recursively from the preliminary core according to the edge clustering coefficient until its density is larger than or equal to a given threshold *T*_*d*_. For an edge (*u*, *v*) ∈ *E*, its edge clustering coefficient [30] ECC(*u*, *v*) is defined as the number of triangles to which (*u*, *v*) belongs, divided by the number of triangles that might potentially include (*u*, *v*), as shown in the following equation:

(2) $$\begin{array}{c} \text{ECC}\left(u,v\right)=\frac{Z_{\left(u,v\right)}}{\min\left\{\deg\left(u\right)-1,\deg\left(v\right)-1\right\}}, \end{array}$$

where *Z*_(*u*,*v*)_ denotes the number of triangles built on the edge (*u*, *v*).

For a preliminary core *C* with den(*C*) < *T*_*d*_, the edge clustering coefficient ECC(*u*, *v*) of each edge (*u*, *v*) connecting the seed vertex *v* and a rest vertex *u* is calculated. Then, vertex *u* with the minimum ECC will be removed from the preliminary core *C*. This operation will be repeated until the density of the new preliminary core *C* is larger than or equal to *T*_*d*_. The preliminary core *C* with den(*C*) ≥ *T*_*d*_ is outputted as a potential protein-complex core.

### 2.2. Generating Potential Dynamic Protein Complexes

After finding the possible protein-complex cores, the key point is how to find the attachments for each core to form protein complexes. One of the most important features of Core-Attachment conception is that the attachment proteins generally bind to the core proteins to carry out cellular functions and are short-lived. Here, all the proteins in *V* which are not included in *P*_*A*_ are considered as potential attachments. On a certain time course, an attachment protein can only participate in one protein complex.

Based on this idea, we first find a best protein-complex core for each potential attachment in the set *P*_*R*_ (*P*_*R*_ = *V* − *P*_*A*_) expressed in a certain time course of the molecular cycle. We say that there is a connection between a protein-complex core and an attachment if there is at least an edge connecting the attachment and one protein in the protein-complex core. An attachment may have connections with multiple protein-complex cores. Here, for each attachment in the set *P*_*R*_ (*P*_*R*_ = *V* − *P*_*A*_) we select a best one with the highest value of “closeness” as its potential protein-complex core. The “closeness” of an attachment *v* to a core *C*_*i*_ is defined as follows:

(3) $$\begin{array}{c} \text{CL}\left(v,C_{i}\right)=\sum_{u\in C_{i}}\text{ECC}\left(v,u\right). \end{array}$$

It is obvious that an attachment *v* tends to be involved in the protein-complex core with the largest CL(*v*, *C*_*i*_). By computing the “closeness” of each attachment protein in *P*_*R*_ to all the possible protein-complex cores, we can get a best corresponding one for each attachment. If the “closeness” of an attachment to all the possible protein-complex cores is equal to 0, this attachment will be ignored. In other words, such a protein in *P*_*R*_ will not be an attachment of any possible protein-complex core.

Then, the potential dynamic protein complexes will be generated based on these correspondences of possible protein-complex cores to attachments in *P*_*R*_. For each time course in the molecular cycle, every protein in *P*_*R*_ will be judged whether it is active or not. If a protein in *P*_*R*_ is active in a certain time course, it will be considered as an attachment and added to its corresponding protein-complex core at this time course. Finally, the protein-complex cores and their attachments with the expressed time course will be outputted. The description of generating potential dynamic protein complexes can be seen in Algorithm 2.

### 2.3. Filtering False Positive Complexes

In the second phase, all the possible dynamic protein complexes are identified by combining the protein-complex cores and their corresponding attachments. For a protein-complex core, only one possible protein complex will be kept if its corresponding attachment groups are the same at different time courses. A protein-complex core may have several different groups of attachments or have no attachments. In the last phase, we will reexamine the possible dynamic protein complexes to filter some false positives. For instance, a protein complex will not provide any information if it only consists of one protein and such protein complex should be removed. According to the formation and function of a protein complex, it should be active in two or more continual time courses. Furthermore, a protein-complex core expressed in all the molecular cycle can be regarded as a protein complex even if it has no attachments.

Based on the above analysis, we use the following rules to filter false positive complexes. (1) A protein complex should include at least two proteins. (2) The attachment proteins should be active in the same time course or in different but adjacent time courses. (3) If the attachments of a possible protein complex do not satisfy the second rule and the protein-complex core involves at least two proteins, the core will be kept as a final protein complex.

The description of algorithm DPC is shown in Algorithm 2. The input of algorithm DPC is a PPI network, gene expression data and two threshold parameters: *T*_*g*_ and *T*_*d*_. The user can get different protein complexes by modifying the values of  *T*_*g*_ and *T*_*d*_. In this paper, the typical threshold values of  *T*_*g*_ and *T*_*d*_ used in previous works [8, 22, 29, 31] were used.

## 3. Experiments and Results

The original protein-protein interaction data of* Saccharomyces cerevisiae*, which was downloaded from the DIP database [32], consists of 4950 proteins and 21,788 interactions. Moreover, gene expression profile, coming from Tu et al. [33], contains 6777 gene products and 36 samples in total, 4858 genes of which are involved in the PPI network of* Saccharomyces cerevisiae*. Our algorithm DPC was applied on the above PPI network and gene expression profile, which generated 766 dynamic protein complexes made up of cores and attachments. In the following subsections, the performance of our algorithm DPC and six other algorithms (MCL [27], CMC [4], SPICi [28], HC-PIN [15], COACH [22], and Core-Attachment [23]) were compared on core analysis, matching with known complexes, function annotation analysis.

### 3.1. Analysis of Protein-Complex Cores

Protein-complex cores are the most important sections of protein complexes. Some attachments bind to the cores to carry out their cellular functions and every protein complex has a unique core. Getting a protein complex from a bad core is likely to generate a protein complex by randomly selecting proteins from a PPI network. Analysis of protein-complex cores is essential for the algorithms based on Core-Attachments assumption.

In our works, protein-complex cores, as the “hearts” of protein complexes, are detected by the integration of PPI network and gene expression profile. Our algorithms DPC generated 550 protein-complex cores in total. Intuitively, a good core should be completely included in known protein complexes. Here, we explore a new indicator, named Core Matching Rate (CMR), to show the performance of cores matched by known complexes. Suppose *K* be a set of known protein complexes and *K*_*i*_ be a known protein complex included in *K*; that is, *K*_*i*_ ∈ *K*. Given a protein-complex core *C*, its CMR is defined as follows:

(4) $$\begin{array}{c} \text{CMR}\left(C\right)=\max\left(\frac{\left|C\cap K_{i}\right|}{\left|C\right|}\right),\;K_{i}\in K, \end{array}$$

where |*C*∩*K*_*i*_| denotes the number of proteins of *C* included in one known proteins complex *K*_*i*_. When a protein-complex core *C* is completely included in a known protein complex *K*_*i*_, CMR(*C*) = 1.

**Figure 1:**
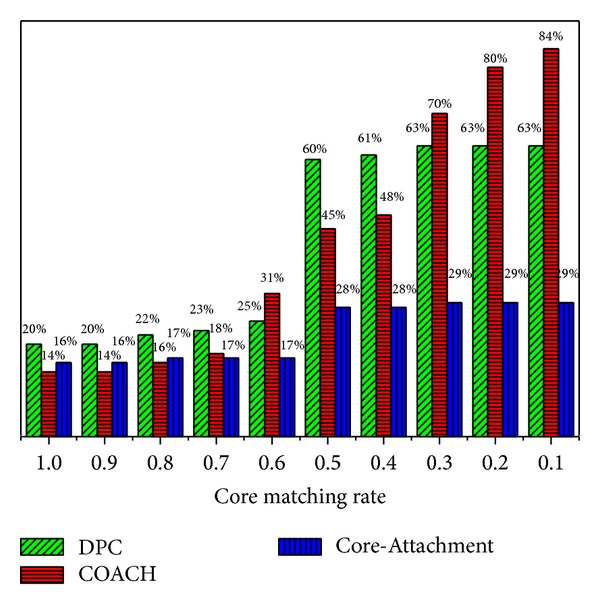
The percentage of protein-complex cores predicted by DPC, COACH, and Core-Attachment with respect to the Core Matching Rate ranging from 1.0 to 0.1.

The known protein complexes were collected from the literature published in Nucleic Acids Research ([34]). There are 532 known protein complexes whose sizes vary from 2 to 81. As COACH and Core-attachment are both developed based on Core-Attachments assumption; we compared the cores produced by these two methods and our algorithm DPC by matching them to the known protein complexes. The comparison results with respect to different core matching rate thresholds from 1.0 to 0.1 are shown in Figure 1. COACH produced 894 protein-complex cores and Core-Sttachment generated 1634 cores. Our algorithm DPC discovered 550 protein-complex cores. From Figure 1 we can see that for the predicted protein-complex cores, about 20% of cores of DPC are completely included in the known complexes, and 14% of COACH and 16% of Core-Attachment are found with CMR(*C*) = 1. When CMR(*C*) ≥ 0.5 (i.e., at least 50% of proteins in the protein-complex core appeared in a known protein complex), the percentage of cores predicted by DPC is 60% which is 15% higher than that predicted by COACH and 32% higher than that predicted by Core-Attachment. From Figure 1 we can see that our algorithm DPC can detect the protein-complex cores more accurately than COACH and Core-Attachment.

**Figure 2:**
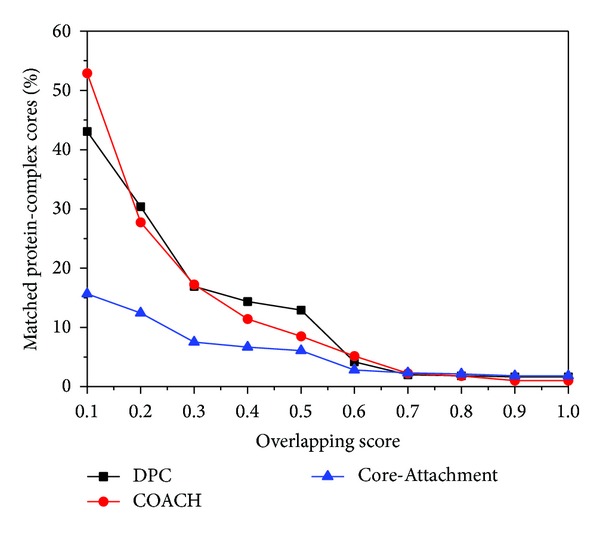
Percentage of matched protein-complex cores predicted by DPC, COACH, and Core-Attachment with respect to various overlapping score thresholds.

To further evaluate the quality of protein-complex cores produced by our algorithm DPC, COACH, and Core-Attachment, we compared them with the cores from Gavin et al. [21] which were obtained by mass spectrometry and bioinformatics analysis. We used overlapping score (OS) which is generally used to validate how effectively a predicted complex (*P*_*C*_) matches a known complex (*K*_*C*_). The formula of *OS*(*P*_*C*_, *K*_*C*_) is shown as follows:

(5) $$\begin{array}{c} \text{OS}\left(P_{C},K_{C}\right)=\frac{{\left|Pc\cap Kc\right|}^{2}}{\left|Pc\right|\times \left|Kc\right|}, \end{array}$$

where |*P*_*C*_∩*K*_*C*_| denotes the number of proteins in the intersection of *P*_*C*_ and *K*_*C*_. |*P*_*C*_| and |*K*_*C*_| represent the number of proteins in *P*_*C*_ and the number of proteins in *K*_*C*_, respectively. Here, not protein complexes but predicted cores are considered. The known cores from Gavin et al. [21] and the cores predicted by DPC will be considered as Kc and Pc, respectively. The matching results with respect to various overlapping score thresholds are shown in Figure 2. The matching results of COACH and Core-Attachment are also shown in Figure 2.

From Figure 2 we can find that when the overlapping score threshold is larger than 0.7, the percentage of matched protein-complex cores predicted by DPC, COACH, and Core-Attachment are almost the same. When the overlapping score is in the range from 0.2 to 0.5, the percentage of the matched cores from DPC is twice that of Core-Attachment and is a little higher than that of COCAH. From the above analysis we can see that the protein-complex cores predicted by DPC are more accurate than those produced by COCAH and Core-Attachments.

### 3.2. Comparison with Known Complexes

To evaluate the effectiveness of our algorithm DPC for detecting protein complexes, we compare the predicted complexes with known protein complexes published in* Nucleic Acids Research* [33]. Here, we use the same scoring scheme (overlapping score) used in [4, 22, 23, 27, 28] to determine how effectively a protein complex is predicted. The definition of overlapping score can be seen in the Subsection 3.1. When the overlapping score of a predicted complex (*P*_*C*_) and a known complex (*K*_*C*_) is equal to 0 (*OS*(*P*_*C*_, *K*_*C*_) = 0), it means that any proteins in *P*_*C*_ do not match to proteins in *K*_*C*_. When OS(*P*_*C*_, *K*_*C*_) = 1, a predicted complex (*P*_*C*_) perfectly matches to a known complex (*K*_*C*_). A predicted complex can be considered as a match to a known complex if their overlapping score is equal to or larger than a specific threshold which is generally set to 0.2 [22, 23, 27].

In addition, two important indicators, specificity and sensitivity, are widely used to evaluate clustering algorithms. Here, we also used them to evaluate our algorithm DPC and some other previous related works. Specificity (*S*_*p*_) is the fraction of the predicted complexes that are matched by the known complexes, divided by the total number of the predicted clusters. Sensitivity (*S*_*n*_) is the fraction of the known complexes that are matched by the predicted clusters among the known complexes. The formula of specificity and sensitivity can be shown as follows:

(6) $$\begin{array}{c} S_{p}=\frac{T_{P}}{T_{P}+F_{P}}\\ S_{n}=\frac{T_{P}}{T_{P}+F_{N}}, \end{array}$$

where *T*_*P*_ (true positive) is the number of the identified complexes that can be matched by one or more known complexes with OS(*P*_*C*_, *K*_*C*_)≥0.2, *F*_*P*_ (false positive) is the number of the detected complexes which can always be matched by each known complex with OS(*P*_*C*_, *K*_*C*_) < 0.2, and *F*_*N*_ (false negative) denotes the number of the known complexes that cannot be matched by any predicted complex with OS(*P*_*C*_, *K*_*C*_) ≥ 0.2. Finally, based on the definitions of specificity (*S*_*p*_) and sensitivity (*S*_*n*_), a comprehensive evaluation indicator *F*-score can be defined as follows:

(7) $$\begin{array}{c} F\text{-score}=\frac{2\times S_{p}\times S_{n}}{S_{p}+S_{n}}. \end{array}$$

The number of predicted complexes and perfected matches (*P*_*m*_), the specificity (*S*_*p*_), sensitivity (*S*_*n*_), and *F*-score of our algorithm DPC and six other previous competing algorithms CMC, MCL, SPICi, HC-PIN, COACH, and Core-Attachment are shown in Table 1. In Table 1, the perfected matches (*P*_*m*_) denote that the number of the predicted protein complexes perfectly matched with known complexes.

As shown in Table 1, our algorithm DPC consistently outperforms the previous algorithms CMC, SPICi, MCL, and HC-PIN on the perfected matches (*P*_*m*_), specificity (*S*_*p*_), sensitivity (*S*_*n*_), and *F*-score. For the Core-Attachment based algorithm, COACH, it produces 894 protein complexes with 16 perfect matches. However, our algorithm DPC has 24 perfect matches with the produce of 766 protein complexes which are less than those of COACH. COACH and DPC have the same specificity, but the sensitivity and *F*-score are a little higher than our algorithm DPC. The specificity, sensitivity, and *F*-score are calculated based on the overlapping score threshold equal to 0.2. However, with the increase of the overlapping score threshold, the predicted complexes of DPC match better to the known complexes than that of COACH. For example, when OS(*P*_*C*_, *K*_*C*_) > 0.5, the percentage of matched complexes of DPC is 9.27% and that of COACH is 9.06%. When OS(*P*_*C*_, *K*_*C*_) > 0.8, the percentage of matched complexes of DPC is more than 3%, however, that of COACH is less than 2%.

For another algorithm Core-Attachment, it produces 1634 protein complexes which are twice more than that generated by our algorithm DPC. The specificity of Core-Attachment is 0.18, which is much lower than our algorithm DPC and other algorithms HC-PIN and COACH. The lower specificity of Core-Attachment indicates that Core-Attachment produces a lot false positives when generating such a large number of protein complexes. Moreover, the comprehensive evaluation indicator *F*-score of our algorithm DPC is much higher than that of Core-Attachment. The former is about 1.6 times the latter.

### 3.3. Functional Annotation Analysis

**Figure 3:**
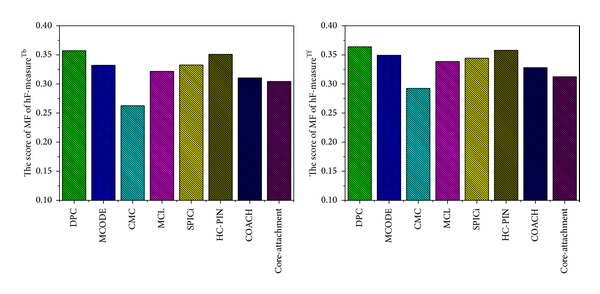
Comparison of DPC, HC-PIN, SPICi, MCL, COACH, Core-Attachment, and CMC on the validations of hF-measure^Tf^ and hF-measure^Tb^.

To get insights on the shared, underlying molecular function of the identified protein complexes, we use Gene Ontology annotations, downloaded from the Saccharomyces Genome Database (SGD) [35], to analyze their enrichments. Several methods have been proposed to evaluate the functional enrichments of predicted complexes. Here, we used the new method, hF-measure [36], we proposed recently. hF-measure is a GO-based functional enrichment analysis method by taking into account the hierarchical organization of functional annotation and the function similarities among proteins. There are two versions of hF-measure: hF-measure^Tf^, a topology-free measurement, and hF-measure^Tb^, a topology-based measurement. We use both hF-measure^Tf^ and hF-measure^Tb^ to evaluate the predicted complexes of our algorithm DPC and those of six other previous competing algorithms: CMC, MCL, SPICi, HC-PIN, COACH, and Core-Attachment. The comparison results are shown in Figure 3.

As shown in Figure 3, the hF-measure^Tf^ and hF-measure^Tb^ of our algorithm DPC are consistently higher than those of six other previous competing algorithms: CMC, MCL, SPICi, HC-PIN, COACH, and Core-Attachment. The comparison results show that no matter whether the topologies are considered, the predicted complexes of DPC have good functional enrichments. In other words, the protein complexes produced by DPC have more chances to implement the same or similar functions and these proteins generally tend to have more interactions among themselves than others.

### 3.4. Detecting Dynamic Protein Complexes

**Figure 4:**
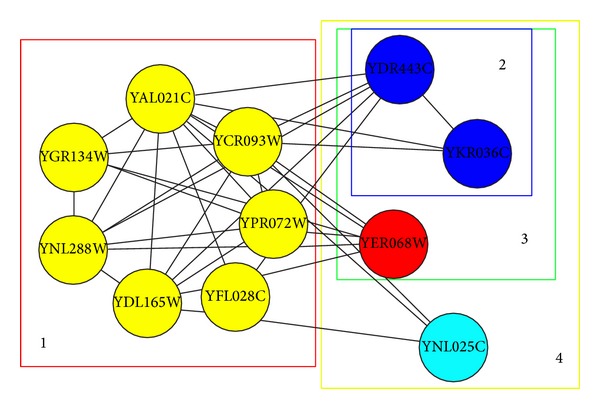
An example of protein complexes identified by DPC (1: a core, 2~4: three sets of attachments).

Distinguished from other previous Core-Attachment based algorithms, our algorithm DPC identified protein complexes by integration of PPI network and gene expression profiles. The implied dynamic information was also contained within the identified protein complexes. The protein-complex cores are a group of proteins which are always active in the molecular cycle and the attachments surrounding the cores are not always active but active in one or multiple time courses. As shown in Figure 4, we give an example to illustrate how the dynamic information was provided by our identified protein complexes.

As shown in Figure 4, the seven proteins in the protein-complex core are always active in all the 36 time courses. However, the three groups of attachments are active in different time courses. The attachments of YDR443C and YKR036C were expressed in the time courses from 13th to 30th. However, YNL025C binds to the core only when it is active in the time courses of 17th and 18th.

**Figure 5:**
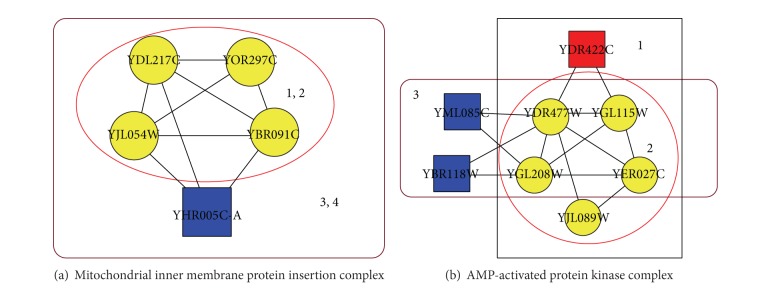
Two examples of known protein complexes recalled by DPC, COACH, and Core-Attachment. (1) known complexes, (2) matched predicted complex of DPC, (3) matched complex of COACH, and (4) matched complex of Core-Attachment.

The integration of gene expression data not only provides implied dynamic information but also contributes to more accurate identification of protein complexes.  Figure 5 shows two examples of known protein complexes recalled by DPC, COACH, and Core-Attachment. As shown in Figure 5(a), the known protein complex “mitochondrial inner membrane protein insertion complex” was perfectly recalled by our algorithm DPC. Meanwhile, both COACH and Core-Attachment provide a larger complex which consists of five proteins YJL054W, YOR297C, YDL217C, YBR091C, and YHR005C-A. The protein complexes identified by DPC, COACH, and Core-Attachment have the same core (YJL054W, YOR297C, YDL217C, and YBR091C). The attachment YHR005C-A was removed from the final complex for it was not active in at least two adjacent time courses. In the last phase of our algorithm DPC, the spurious attachments will be removed according to our filtering rules. Thus, our algorithm DPC can use gene expression data to identify more accurate complexes.

Figure 5(b) shows an example of known protein complex (“AMP-activated protein kinase complex”) which was partly recalled by DPC, COACH, and Core-Attachment. The “AMP-activated protein kinase complex” consists of six proteins. Out of the six proteins, five proteins YER027C, YDR477W, YGL208W, YJL089W, and YGL115W were recalled by our algorithm DPC. The overlapping score OS(Pc, Kc) is 0.83 between the known complex and the predicted complex of DPC. However, the best overlapping score OS(Pc, Kc) is 0.44 between the known complex and a predicted complex of COACH. The best matched complex of COCAH recalled only four members of the known complexes and predicted two false positives. Core-Attachment cannot find a complex matching with the known complex with OS(Pc,Kc) ≥ 0.1. The best match of predicted complex from Core-Attachment consists of 23 proteins and only has one protein included in the known complex of “AMP-activated protein kinase complex.”

## 4. Conclusion

In postgenomic era, one of the key topics in system biology is to recognize life activity with a cell by protein interactions and protein complexes. Many computational algorithms have been proposed to identify protein complexes from static protein-protein interaction data. In reality, proteins and interactions between proteins are dynamic in cellular life. Identifying dynamic protein complexes has become an essential and challenging task in the system biology. In this paper, a novel algorithm DPC has been proposed to identify dynamic protein complexes by integrating PPI network and gene expression data. The algorithm DPC has been developed based on the Core-Attachments assumption that the always active proteins involved in the core and some other not always active proteins bind to the core dynamically.

The effectiveness of our algorithm DPC has been tested on the PPI network and gene expression of* Saccharomyces cerevisiae*. The experimental results based on matching with known protein complexes and cores have shown that our algorithm DPC can predict more accurate cores than COACH and Core-Attachment. In addition, our algorithm DPC outperforms the previous algorithms CMC, SPICi, MCL, and HC-PIN on the number of perfect matches, specificity and sensitivity and *F*-score. Moreover, all the identified protein complexes of DPC, CMC, MCL, SPICi, HC-PIN, COACH, and Core-Attachment have been validated on the functional enrichments. The latest GO based methods hF-measure^Tf^ and hF-measure^Tb^ are used. The experimental results have shown that the hF-measure^Tf^ and hF-measure^Tb^ of the predicted complexes identified by our algorithm DPC are consistently higher than that of six other previous competing algorithms: CMC, MCL, SPICi, HC-PIN, COACH, and Core-Attachment which indicates that no matter whether the topologies are considered, the predicted complexes of DPC have good functional enrichments. In this paper, only gene expression is integrated. Actually, more biological information, such as subcellular localization and biological processes, can also be integrated to help identify protein complexes more accurately. This will be one of our future works.

## Figures and Tables

**Algorithm 1:**
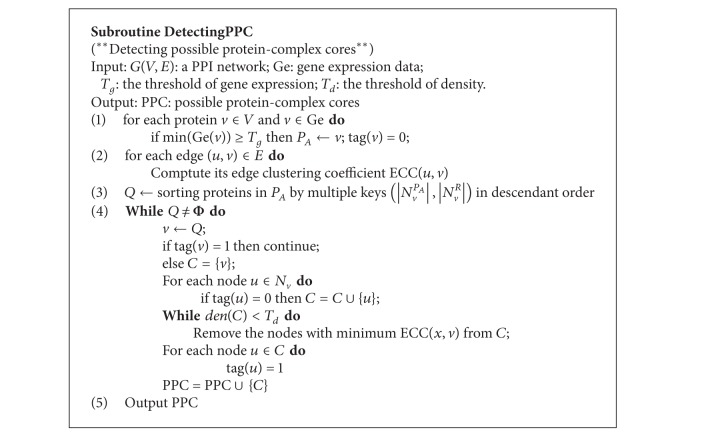
The description of subroutine for detecting possible protein-complex cores.

**Algorithm 2:**
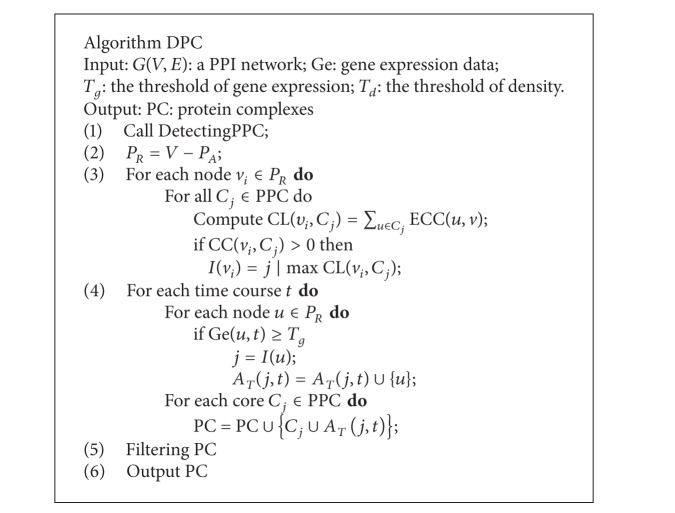
The description of algorithm DPC.

**Table 1:** Comparison of DPC and six other algorithms: CMC, MCL, SPICi, HC-PIN, COACH, and Core-Attachment on the number of predicted complexes and perfected matches (*P*_*m*_), specificity (*S*_*p*_), sensitivity (*S*_*n*_), and *F*-score.

Algorithms	Number	*P*_*m*_	*S*_*n*_	*S*_*p*_	*F*-score
DPC	**766**	**24**	**0.51**	**0.37**	**0.43**
CMC	981	3	0.37	0.18	0.24
MCL	932	16	0.39	0.20	0.26
SPICi	552	9	0.29	0.24	0.26
HC-PIN	274	19	0.28	0.46	0.35
COACH	894	16	0.57	0.37	0.45
Core-Attachment	1634	35	0.58	0.18	0.27
